# Detection of Aerosolized *Mycobacterium tuberculosis* DNA From Adults Being Investigated for Pulmonary Tuberculosis via an Electrostatic Sampler in a South African Primary Care Setting

**DOI:** 10.1093/ofid/ofaf593

**Published:** 2025-09-24

**Authors:** Jay Achar, Rouxjeane Venter, Jamie van Schalkwyk, Zandile Booi, Zama Mahlobo, Zaida Palmer, Nuno Rufino de Sousa, Knut Lönnroth, James A Seddon, Antonio Gigliotti Rothfuchs, Grant Theron

**Affiliations:** Department of Global Public Health, Karolinska Institutet, Stockholm, Sweden; DSI-NRF Centre of Excellence for Biomedical Tuberculosis Research, South African Medical Research Council Centre for Tuberculosis Research, Division of Molecular Biology and Human Genetics, Faculty of Medicine and Health Sciences, Stellenbosch University, Cape Town, South Africa; DSI-NRF Centre of Excellence for Biomedical Tuberculosis Research, South African Medical Research Council Centre for Tuberculosis Research, Division of Molecular Biology and Human Genetics, Faculty of Medicine and Health Sciences, Stellenbosch University, Cape Town, South Africa; DSI-NRF Centre of Excellence for Biomedical Tuberculosis Research, South African Medical Research Council Centre for Tuberculosis Research, Division of Molecular Biology and Human Genetics, Faculty of Medicine and Health Sciences, Stellenbosch University, Cape Town, South Africa; DSI-NRF Centre of Excellence for Biomedical Tuberculosis Research, South African Medical Research Council Centre for Tuberculosis Research, Division of Molecular Biology and Human Genetics, Faculty of Medicine and Health Sciences, Stellenbosch University, Cape Town, South Africa; DSI-NRF Centre of Excellence for Biomedical Tuberculosis Research, South African Medical Research Council Centre for Tuberculosis Research, Division of Molecular Biology and Human Genetics, Faculty of Medicine and Health Sciences, Stellenbosch University, Cape Town, South Africa; DSI-NRF Centre of Excellence for Biomedical Tuberculosis Research, South African Medical Research Council Centre for Tuberculosis Research, Division of Molecular Biology and Human Genetics, Faculty of Medicine and Health Sciences, Stellenbosch University, Cape Town, South Africa; Department of Microbiology, Tumor and Cell Biology, Karolinska Institutet, Stockholm, Sweden; Department of Global Public Health, Karolinska Institutet, Stockholm, Sweden; Desmond Tutu TB Centre, Department of Paediatrics and Child Health, Stellenbosch University, Cape Town, South Africa; Department of Infectious Disease, Imperial College London, London, UK; Department of Microbiology, Tumor and Cell Biology, Karolinska Institutet, Stockholm, Sweden; DSI-NRF Centre of Excellence for Biomedical Tuberculosis Research, South African Medical Research Council Centre for Tuberculosis Research, Division of Molecular Biology and Human Genetics, Faculty of Medicine and Health Sciences, Stellenbosch University, Cape Town, South Africa

**Keywords:** cough-generated aerosol, diagnosis, electrostatic sampler, *Mtb* aerosolization, *Mycobacterium tuberculosis*

## Abstract

**Background:**

Non–sputum-based diagnosis of tuberculosis is a public health priority. Little is known about the feasibility of detecting *Mycobacterium tuberculosis* (*Mtb*) complex DNA in respiratory aerosols in primary care, its diagnostic value, and clinical and microbiological characteristics associated with detection.

**Methods:**

We recruited symptomatic adults self-presenting to South African primary care clinics with a sputum Xpert MTB/RIF Ultra (Ultra) result. Cough aerosols were collected on-site by the TB Hotspot Detector, a novel electrostatic aerosol sampler, and tested by Ultra. Environmental and laboratory controls were collected. Predictors of aerosol *Mtb* DNA (AMD) detection were assessed.

**Results:**

Among 137 participants, 71 (52%) had medium or high sputum Ultra semiquantitative results and 34 (25%) had negative results. When compared with sputum Ultra detection, AMD detection sensitivity and specificity were 46.6% (95% CI, 42.5%–50.7%) and 76.5% (95% CI, 70.4%–82.5%), respectively. Sensitivity was higher in people with a sputum Ultra semiquantitation category of high (56.9%; 95% CI, 51.1%–62.7%). Factors associated with AMD detection were male sex with a sputum Ultra semiquantitative result of medium or greater (adjusted risk ratio, 3.26; 95% CI, 1.11–9.55; *P* = .024) and a reported fever (adjusted risk ratio, 0.58; 95% CI, .29–1.07; *P* = .099). Sputum to aerosol ratios were ≥0.75 in 3 participants, suggesting a high capacity to expel *Mtb* DNA. Despite rigorous decontamination, AMD was detected from 30% of environmental samples, highlighting the TB Hotspot Detector's potent sampling capability and potential nosocomial transmission risks.

**Conclusions:**

Electrostatic aerosol sampling is feasible in primary care to detect people with infectious tuberculosis. Deployment of this and other practical aerosol-sampling tools might help to characterize predictors of tuberculosis transmission.

## Teaser

Collecting aerosol from people investigated for tuberculosis in primary care is challenging. Using electrostatic sampling, we detected *Mycobacterium tuberculosis* DNA from 48 (46%) of 103 people diagnosed with tuberculosis. This approach could facilitate better at-scale characterization of infectiousness in diverse settings.

The World Health Organization estimated that 10.8 million people became ill and 1.25 million people died from tuberculosis (TB) in 2023 [1]. In the same year, 8.2 million cases were notified by national programs, representing a 24% detection gap. Reducing TB incidence by 90% in 2030 as compared with 2015 is a core aim of the End TB strategy [2]. To disrupt the TB transmission cycle, earlier diagnosis, especially in highly infectious cases, is crucial.

The search for non–sputum-based TB diagnostic tests is motivated by sputum scarcity (defined by an inability to produce sputum for testing) in a significant proportion of people being investigated for TB. Sputum scarcity has been estimated to affect up to 7.6% of participants in country prevalence surveys [3] and 18% of people with HIV being investigated for TB [4]. To overcome this barrier to diagnosis, exhaled breath has been proposed as a diagnostic specimen and has shown promise through the application of filters to face masks [5] and blow tubes [6].

Detection of aerosolized *Mycobacterium tuberculosis* (*Mtb*) may provide information about infectiousness. First described in the late 19th century [7], aerosolization of *Mtb* is known to be a critical step in transmission. Aerosol detection is more predictive of transmission than routinely used proxies such as sputum smear microscopy [8]. Among exposed household contacts, aerosolized *Mtb* detection from the source patient was positively associated with new *Mtb* infections [9] and incident cases of TB disease [10]. Despite more recent development of aerosolized *Mtb* detection devices [11], relatively few data exist to estimate individual infectiousness. Available systems for sampling aerosols require highly specialized equipment and staff and a laboratory for sample analysis, restricting their use to a handful of research settings [11, 12].

Broadening aerosol sampling to encompass diverse patient populations across multiple geographic regions would strengthen the evidence base for developing targeted interventions to reduce transmission. Aerosol testing may also generate valuable data to estimate the contribution of different groups to community transmission—for example, people with negative sputum test results and asymptomatic people with laboratory-confirmed TB [13–16].

In this study, we describe the detection of *Mtb* DNA from cough aerosol in primary care clinics using an electrostatic air sampler that is portable, quiet, and compact (TB Hotspot Detector [THOR]) paired with a semiautomated nucleic acid amplification test (Xpert MTB/RIF Ultra; henceforth, Ultra). We focused on symptomatic people to justify future research on special groups, including those without recognizable symptoms and where sputum scarcity is frequent.

## METHODS

### Participant Flow

Adults (≥18 years) presenting to 4 primary health clinics in Cape Town, South Africa, with symptoms of pulmonary TB between April and December 2024 were enrolled. Participants must have had sputum Ultra testing within 7 days prior to inclusion. Until July 2024, inclusion was restricted to people with sputum medium or high Ultra semiquantitative results; after which, sputum results were not used to define eligibility (Supplementary Figure 1).

People with hemoptysis in the preceding 7 days were excluded to avoid the risk of cough-induced hemoptysis, a potentially life-threatening condition. People receiving TB treatment in the prior 60 days were also excluded.

### Aerosol Sampling

**Figure 1.**
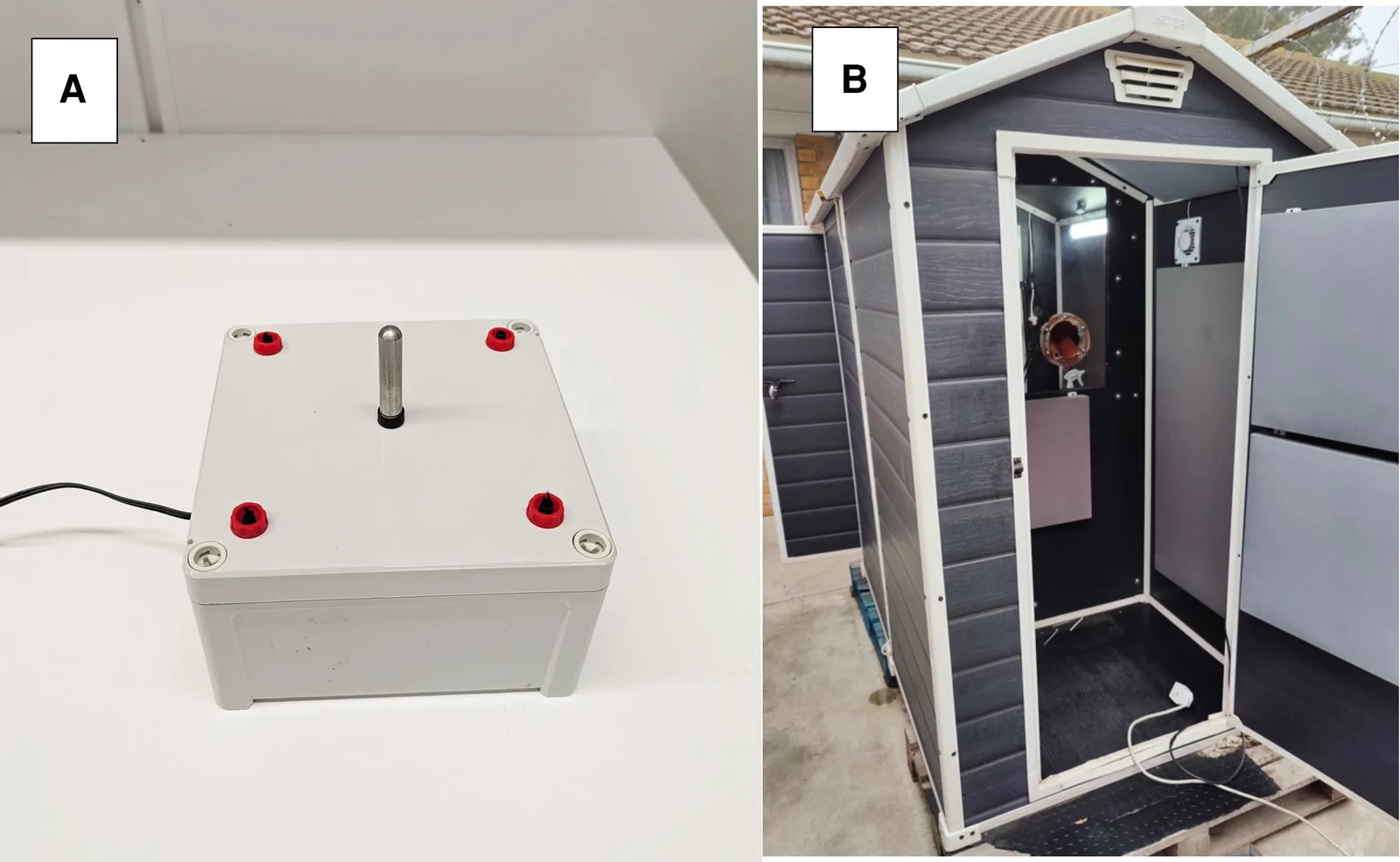
*A*, The TB Hotspot Detector (THOR) measures 13 × 13 × 7.5 cm (L × W × H) and weighs 0.4 kg. Replaceable carbon brush ionizers are mounted on the outside of the device as red ports. A removeable stainless-steel collector piece is attached to THOR via an electrically grounded magnet on top of the device. It is powered by any standard AC power supply (10–240 V) or a portable battery. THOR has no moving parts and operates silently at lower power. *B*, The sampling booth measured 1.1 × 0.9 × 1.9 m (L × W × H), creating an internal volume of 1.9 m^3^ with a single door for entry and exit. During sampling, participants stood inside against a wall, with THOR on a small table against the opposite wall approximately 1 m away.

Participant aerosol was collected by the electrostatic air sampler THOR [17]. Measuring 13 × 13 × 7.5 cm (L × W × H) and weighing 0.4 kg, THOR is quiet, compact, portable, and lightweight (Figure 1*A*). When connected to a power source, THOR uses corona discharge to ionize particles in the surrounding air. Charged particles are accelerated toward and precipitate on the surface of a grounded collector piece: a stainless-steel rod measuring 3.5 cm in height and 0.88 cm in diameter. After sample collection, THOR is switched off, and the collector piece is aseptically removed and stored in a sterile microcentrifuge tube for transport to the laboratory. To verify correct operation, power consumption was measured from portable battery packs when THOR was activated.

**Figure 2.**
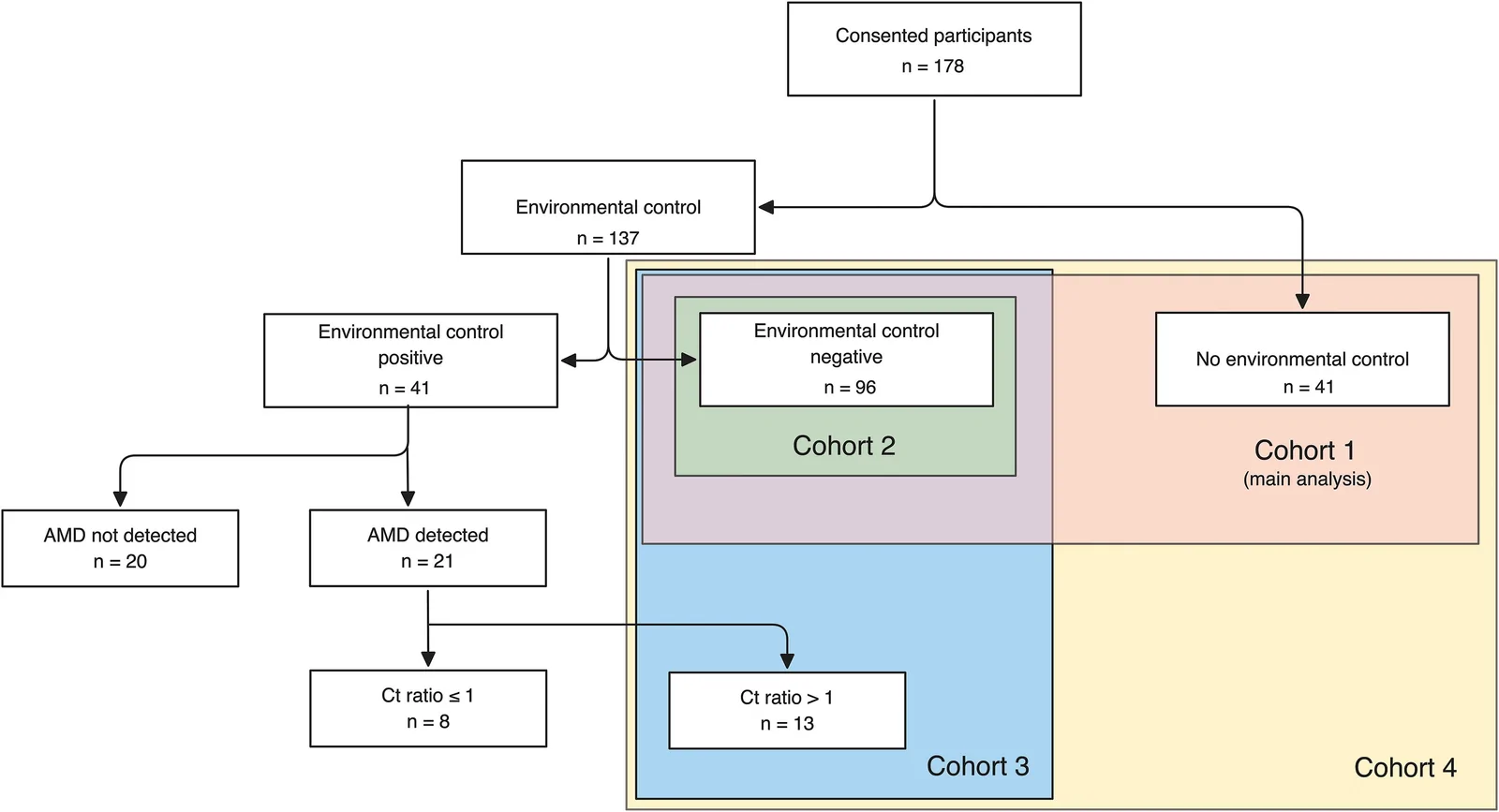
Study flow and definition of main analysis cohort and sensitivity analysis cohorts. Overall, 178 people fulfilled the inclusion criteria and consented to participate. An environmental control result was available for 137 (77%), of which 41 (30%) were positive. Of these participants, cough AMD was detected from 21 (51%), with 13 (62%) having lower Xpert MTB/RIF Ultra IS*1081/*IS*6110* probe C_T_ values from cough aerosol samples than environmental aerosol samples, signifying the detection of larger quantities of AMD. Four cohorts were defined. Cohort 1 (137/178) included participants with a negative or no environmental control result, and this was used for the main analysis. Cohort 2 (96/178) included only participants with a negative environmental control. Cohort 3 (109/178) included all participants from cohort 2 alongside participants with a positive environmental control but a C_T_ value ratio >1, reflecting additional DNA detection from cough aerosol samples. Cohort 4 (150/178) included all participants in the other 3 cohorts. Abbreviations: AMD, aerosol *Mycobacterium tuberculosis* DNA; C_T_, cycle threshold.

Two consecutive aerosol samples were collected from each participant. For each collection, unmasked participants stood facing THOR at a distance of approximately 80 cm; from there, they coughed as frequently as possible, at least 10 times, inside a dedicated sampling booth at each clinic for 5 minutes, with the door closed and THOR switched on. Cough-generated aerosol passively dispersed inside the sampling booth since no mouthpiece was used. After the participant exited the booth, the door was closed, and THOR continued sampling for 5 additional minutes.

Sampling booths measured 110 × 90 × 190 cm (L × W × H), creating an internal volume of 1.9 m^3^ (Figure 1*B*). Each booth was fitted with 4 wall-mounted extractor fans to provide active ventilation between participants. After each participant completed 2 sample collections and at the end of each day, sampling booths and THOR devices were disinfected with 70% ethanol antilint wipes and aerated with extractor fans working for at least 15 minutes. From July 2024, two environmental control samples were taken from inside the booth with the same sampling procedures (10-minute sampling, door closed) prior to each participant sampling. Laboratory control samples were tested each time that aerosol samples were processed and tested.

### Laboratory Processing

Phosphate-buffered saline (450μL; Merck KGaA) containing 0.025% Tween 80 (Sigma-Aldrich) was added to the first microcentrifuge tube containing a collector piece upon receipt at the laboratory, allowed to incubate at room temperature for 2 minutes, and vortexed for 1 minute. The collector piece was removed with a magnet, and the sample-buffer mix from the first collector piece was added to the second collector piece from the same participant and the incubation and vortexing repeated. The second collector piece was removed and the final volume made up to 2 mL with Tris-EDTA buffer (Thermo Fisher Scientific) before being loaded into the Ultra cartridge (version 2; Cepheid) and tested according to the manufacturer's instructions. Xpert sample reagent was omitted to optimize detection.

### Definitions

The minimum cycle threshold (C_T_) is the lowest nonzero *rpoB* C_T_ value reported by Ultra. Semiquantitative categorization, defined by the manufacturer, uses this value to provide automated estimates of *Mtb* cell count in laboratory reports [18]. Ultra trace results arise exclusively from IS*1081/*IS*6110* amplification, the copy number of which varies across strains. To permit inclusion of patients with an aerosol or sputum trace result, environment to aerosol and sputum to aerosol IS*1081/*IS*6110* C_T_ ratios were calculated for each individual. As lower C_T_ values reflect higher DNA (and lower denominators yield higher ratios), ratios >1 signify more *Mtb* DNA in aerosol.

Sputum Ultra semiquantitative results were grouped into high and medium (≥medium); low, very low, and trace (≤low); and negative.

Cohort 1 (main analysis cohort) comprised all participants with a negative environmental control or with no environmental control recorded (Figure 2). Cohort 2 consisted only of participants with a negative environmental control, while cohort 3 extended cohort 2 by including participants with an environment to aerosol IS*1081/*IS*6110* C_T_ ratio >1, reflecting detection of additional *Mtb* DNA in the cough aerosol sample. Cohort 4 included all participants from cohorts 1, 2 and 3.

### Analysis

Participant characteristics are presented as count (percentage) or median (IQR) for the main cohort and after stratification by sputum Ultra semiquantitative result. Aerosol *Mtb* DNA (AMD) detection was dichotomized and presented as count (percentage). The sensitivity and specificity of AMD detection were calculated by using sputum Ultra as the reference standard.

Sputum to aerosol IS*1081/*IS*6110* C_T_ ratio was calculated only for participants with negative environmental controls (cohort 2) to avoid positive bias in the aerosol C_T_ value. Calculation of the environment to aerosol C_T_ ratio was restricted to participants with positive AMD detection and in whom preceding environmental sampling was positive.

Bivariable associations between baseline characteristics and AMD detection were described with crude risk ratios (RRs), 95% CIs, and *P* values. Multivariable modified Poisson regression [19] was used to adjust for confounding. Age, sex, and HIV status were assessed for inclusion a priori. Characteristics with *P* < .1 on bivariable analysis with AMD detection were also assessed for inclusion. Predictor selection employed a forward stepwise approach by using the Akaike information criterion to guide sequential addition. A complete-case approach was used since no missing values were recorded. Sputum Ultra minimum C_T_ was excluded from further evaluation due to missing values from trace and negative results.

Sensitivity analyses including participants from cohorts 2 to 4 were conducted. Statistical analyses were performed with R software version 4.4.1.

## RESULTS

### Study Population and Participant Characteristics

Of 178 sampled participants (Figure 2), environmental controls were collected for 137 (77%), of which 96 (70%) were negative and included alongside 41 (23%) of 178 with no environmental control (cohort 1, n = 137). All laboratory controls were negative. All characteristics of participants by availability of environmental control and environmental control results are available in Supplementary Table 1.

Among the 137 participants in cohort 1, the median age was 36 years (IQR, 29–46), and 78 (57%) were men. Thirty-one (23%) participants were underweight (body mass index ≤18.5), and 57 (42%) had HIV. Thirty-two (56%) participants with HIV were receiving antiretroviral therapy. Preselection by sputum Ultra resulted in 102 (75%) participants having *Mtb* DNA detected in their sputum and 71 (52%) having an Ultra semiquantitative result ≥medium (Table 1). When participants were compared by sputum Ultra semiquantitative result, a higher median body mass index was recorded in people with negative sputum as compared with those with positive sputum, while the median total cough count and median Karnofsky score were higher in people with lower or negative Ultra semiquantitative results (Supplementary Table 2).

**Table 1.** Participant Clinical and Demographic Characteristics (n = 137)

	Median (IQR) or No. (%)
Age, y	36 (29–46)
Sex	
Female	59 (43)
Male	78 (57)
Body mass index, kg/m^2^	21.3 (18.8–24.7)
≤18.5	31 (23)
>18.5	106 (77)
HIV status	
Negative	80 (58)
Positive	57 (42)
ART: no	25 (44)
ART: yes	32 (56)
History of TB	
No	88 (64)
Yes	49 (36)
Smoking history	
Never	61 (45)
Ex-smoker	20 (15)
Current smoker	56 (41)
Total cough count	75 (50–103)
Sputum Ultra minimum C_T_	18.3 (17.5–21.1)
Unavailable^a^	38
Sputum Ultra result	
*Mtb* detected	103 (75)
*Mtb* not detected	34 (25)
Sputum Ultra semiquantitative result	
High	51 (37)
Medium	20 (15)
Low	18 (13)
Very low	10 (7.3)
Trace	4 (2.9)
Negative	34 (25)
Karnofsky score	
50	1 (0.7)
60	3 (2.2)
70	9 (6.6)
80	48 (35)
90	61 (45)
100	15 (11)
Cough	
No	6 (4.4)
Yes	131 (96)
Fever	
No	94 (69)
Yes	43 (31)
Weight loss	
No	31 (23)
Yes	106 (77)
Night sweats	
No	45 (33)
Yes	92 (67)

Abbreviations: ART, antiretroviral therapy; C_T_, cycle threshold; *Mtb*, *Mycobacterium tuberculosis*; TB, tuberculosis; Ultra, Xpert MTB/RIF Ultra.

^a^Unavailable: all missing minimum Ct values were from trace or negative sputum Ultra results.

### AMD Detection

With sputum Ultra as a reference standard, overall sensitivity and specificity of AMD detection were 46.6% (95% CI, 42.5%–50.7%) and 76.5% (95% CI, 70.4%–82.5%), respectively. Sensitivity was higher in people with high (56.9% ; 95% CI, 51.1%–62.7%) or medium (60%; 95% CI, 50.9%–69.1%) sputum Ultra semiquantitative results than in people with low (22.2%; 95% CI, 14.0%–30.4%) or very low (30%; 95% CI, 17.9%–42.1%) results. AMD was detected in 8 (23.5%) people with negative sputum Ultra results (Supplementary Table 3).

AMD detection was associated with all measures of sputum bacillary load (Table 2). When AMD was detected, most aerosol Ultra semiquantitative results were very low (n = 14, 25%) or trace (n = 39, 70%; Supplementary Table 4). To prevent bias through environmental TB DNA detection, sputum to aerosol IS*1081/*IS*6110* C_T_ ratio calculation was restricted to 22 participants in cohort 2 with positive sputum and AMD Ultra results. In 3 participants, a ratio ≥0.75 was recorded, suggesting a high capacity to expel pulmonary bacterial DNA (Supplementary Figure 2).

**Table 2.** Aerosolized *Mtb* DNA Detection and Crude Associations With Participant Characteristics

	Aerosol *Mtb* DNA Detected, Median (IQR) or No. (%)			Crude		
	No.	Yes (n = 56)	No (n = 81)	RR	95% CI^a^	*P* Value
Age, y	137	40 (30–48)	35 (27–45)	1.01	.99–1.03	.3
Sex	137					
Female		17 (29)	42 (71)	…	…	
Male		39 (50)	39 (50)	1.74	1.09–2.75	.019
Body mass index, kg/m^2^	137	21.2 (19.1–25.3)	21.4 (18.7–24.3)	1.02	.98–1.06	.3
≤18.5		13 (42)	18 (58)	…	…	
>18.5		43 (41)	63 (59)	0.97	.60–1.56	.9
HIV status	137					
Negative		36 (45)	44 (55)	…	…	
Positive		20 (35)	37 (65)	0.78	.51–1.20	.3
History of TB	137					
No		37 (42)	51 (58)	…	…	
Yes		19 (39)	30 (61)	0.92	.60–1.42	.7
Smoking history	137					
Never		24 (39)	37 (61)	…	…	
Ex-smoker		9 (45)	11 (55)	1.14	.64–2.05	.7
Current smoker		23 (41)	33 (59)	1.04	.67–1.63	.9
Total cough count	137	61 (49–92)	82 (53–107)	0.99	.99–1.00	.038
Sputum Ultra minimum C_T_	99	18 (17–23)	24 (18–40)	0.93	.86–1.00	.052
Unavailable^b^		8	30			
Sputum Ultra result	137					
*Mtb* detected		48 (47)	55 (53)	…	…	
*Mtb* not detected		8 (24)	26 (76)	0.50	.26–.96	.038
Sputum Ultra category	137					
≤Low/negative		15 (23)	51 (77)	…	…	
≥Medium		41 (58)	30 (42)	2.54	1.56–4.15	<.001
Karnofsky score	137					
>80		26 (34)	50 (66)	…	…	
≤80		30 (49)	31 (51)	1.44	.96–2.16	.080
Cough	137					
No		1 (17)	5 (83)	…	…	
Yes		55 (42)	76 (58)	2.52	.41–15.5	.3
Fever	137					
No		44 (47)	50 (53)	…	…	
Yes		12 (28)	31 (72)	0.60	.35–1.01	.056
Weight loss	137					
No		10 (32)	21 (68)	…	…	
Yes		46 (43)	60 (57)	1.35	.77–2.35	.3
Night sweats	137					
No		17 (38)	28 (62)	…	…	
Yes		39 (42)	53 (58)	1.12	.72–1.76	.6

Abbreviations: C_T_, cycle threshold; *Mtb*, *Mycobacterium tuberculosis*; TB, tuberculosis; Ultra, Xpert MTB/RIF Ultra.

^a^95% CIs calculated with robust standard errors.

^b^Unavailable: all unavailable minimum Ct values were from trace or negative sputum Ultra results.

### Factors Associated With AMD Detection

In unadjusted analysis, the following were positively associated with AMD detection: male sex (RR, 1.74; 95% CI, 1.09–2.75; *P* = .019), Karnofsky score ≤80 (RR, 1.44; 95% CI, .96–2.16; *P* = .080), and sputum Ultra ≥medium (RR, 2.54; 95% CI, 1.56–4.15; *P* < .001). Total cough count (RR, 0.99; 95% CI, .99–1.00; *P* = .038) was negatively associated with AMD detection. There was also moderate evidence of a crude negative association between reported fever (RR, 0.60; 95% CI, .35–1.01; *P* = .056) and AMD detection (Table 2).

Results from the multivariable modified Poisson regression model are presented in Table 3 and Supplementary Figure 3. In addition to sex and sputum Ultra semiquantitative result, reported fever was included in the adjusted model. Inclusion of age, HIV status, total cough count, and Karnofsky score did not improve model fit, and these variables were therefore excluded. An interaction between sex and sputum Ultra semiquantitative result improved model fit and was included.

**Table 3.** Adjusted Associations Between Participant Characteristics and Aerosolized *Mtb* DNA Detection

	aRR	95% CI^a^	*P* Value
Sex: sputum Ultra semiquantitative result			
Female: ≤low/negative	…	…	
Female: ≥medium	1.89	.53–6.7	.571
Male: ≤low/negative	1.13	.3–4.26	.996
Male: ≥medium	3.26	1.11–9.55	.024
Reported fever			
No	…	…	
Yes	0.58	.29–1.07	.099

Abbreviations: aRR, adjusted risk ratio; *Mtb*, *Mycobacterium tuberculosis*; Ultra, Xpert MTB/RIF Ultra.

^a^95% CIs calculated with robust standard errors.

### Interaction Between Sex and Sputum Bacillary Load on AMD Detection

When compared with women with sputum Ultra ≤low/negative results, the adjusted risk of AMD detection was highest in men with sputum Ultra ≥medium (adjusted RR [aRR], 3.26; 95% CI, 1.11–9.55; *P* = .024). There was no evidence of an association with AMD detection in women with sputum Ultra ≥medium (aRR, 1.89; 95% CI, .53–6.7; *P* = .571) or men with sputum ≤low/negative (aRR, 1.13; 95% CI, .3–4.26; *P* = .996). After adjusting for sex and sputum Ultra semiquantitative result, weak evidence of a negative association between reported fever and AMD detection remained (aRR, 0.58; 95% CI, .29–1.07; *P* = .099).

Among 57 of 137 participants with HIV, AMD was detected from 20 (35%; Table 2). From 41 positive environmental controls (Figure 2), subsequent participant AMD detection occurred in 21 (51%). AMD IS*1081/*IS*6110* C_T_ was lower in participant samples than in environmental samples in 13 of 21 (62%), indicating AMD production by the participant (Supplementary Figure 4).

Findings from sensitivity analyses of cohorts 2 to 4 were comparable to findings from the main analysis. However, there was more evidence of a crude negative association between positive HIV status and AMD detection in cohort 2 (RR, 0.43; 95% CI, .20–.91; *P* = .027) and cohort 3 (RR, 0.46; 95% CI, .25–.84; *P* = .012; Supplementary Table 5).

## DISCUSSION

The electrostatic air sampler THOR demonstrated that AMD detection in primary care is feasible by recording positive results from almost half of included participants presenting with pulmonary TB symptoms in an endemic setting.

This study generated several key findings. Detection was more frequent in participants with higher sputum bacillary load, but the significance of detection in the approximately one-fifth of people with low or absent sputum bacilli is unknown. There was evidence of an interaction between sex and sputum bacillary load, and lower risk of AMD detection in people reporting fever. In approximately one-third of cases, environmental controls from booths were positive prior to participants entering, despite stringent cleaning procedures.

Published clinical studies measuring aerosolized *Mtb* have generally used either the Cough Aerosol Sampling System (CASS) or the Respiratory Aerosol Sampling Chamber (RASC) [11, 12]. The CASS and an updated version of the RASC [20] direct exhaled air or cough using mouth or head pieces and vacuum pumps onto detection devices, allowing for larger droplet deposition and minimizing environmental desiccation that occurs during aerosol suspension. While this optimizes detection, it may overestimate infectiousness. In contrast, THOR sampled ambient air within the sampling booth, more closely mimicking the circumstances of an exposure event.

Comparing detection data from studies that utilized the CASS or RASC systems is challenging due to differences in study populations and sampling durations. CASS studies have predominantly included participants with positive sputum smear microscopy or culture and have sampled participants for around 10 minutes. Aerosol detection increased from approximately 25% in earlier studies [11, 21, 22] to 65% in more recent studies [9, 23], partially through the exclusion of participants receiving treatment [8]. Studies performed with the RASC describe higher aerosolized *Mtb* detection (70%–92%) [24, 25], with the highest detection described in recent studies [25, 26] that employed droplet digital polymerase chain reaction (PCR), a research technique with high precision.

With THOR, we detected AMD in approximately 60% of participants with high and medium sputum Ultra semiquantitative results, similar to findings from recent CASS studies [9, 23]. Detection was lower in people with less or no sputum *Mtb* DNA and lower than in comparable participants in RASC studies that used droplet digital PCR [26, 27]. The significance of detection of AMD in people with negative sputum results is uncertain. While detection may represent fluctuation in clinical disease, sampling stochasticity, or airway colonization, it may be consistent with recent RASC studies and epidemiologic research from high-incidence settings, which hypothesize significant community transmission originating from this group [28].

The detection of *Mtb* DNA in environmental samples from TB clinics was described in a South African study where 10% to 42% of samples were positive [29, 30]. The positive environmental controls in our study confirm the potent sampling capability of THOR, its potential role as an environmental air-monitoring device, and the high risk of *Mtb* exposure in clinics within high-incidence settings [31], therefore highlighting the need for additional optimization of cleaning procedures associated with its use.

We identified a complex association between sex and AMD detection. Unadjusted results found men to have 1.5 times the risk of AMD detection than women and people with higher sputum Ultra semiquantitative results to have 2.5 times the risk of AMD detection than those with lower or negative results. After adjusting for self-reported fever, an interaction between sex and sputum *Mtb* bacillary load illustrated a stronger effect of sex in people with higher detection of sputum *Mtb* DNA such that men in this group had a 3-fold higher risk of AMD detection than women with low or negative sputum Ultra results. Our finding of a positive association between sputum *Mtb* load and aerosol detection is consistent with previous transmission studies showing that higher bacterial burden—as measured by smear microscopy grade [32], liquid culture time to positivity [33], and PCR C_T_ values [34, 35]—is associated with increased transmission risk. Sex differences in respiratory tract aerosolized particle release have not been described, but in people diagnosed with TB, a crude association between sex and aerosolized *Mtb* detection was noted in 1 large South African study [22] where women had 46% lower odds of aerosolized *Mtb* detection than men. Furthermore, molecular epidemiologic findings from the Netherlands have described more secondary cases originating from male sources than female and that cases due to recent transmission were mainly linked to male source cases [36]. Our findings may reflect structural and behavioral factors that result in worse health outcomes in men as compared with women [37], thereby underscoring the importance of diagnosing TB in men more effectively. The negative association between participant-reported fever and AMD detection is also consistent with previous studies where the presence of fewer symptoms has been associated with greater aerosol detection [22], possibly due to better physical health permitting stronger cough maneuvers.

Although our study shows that THOR can be used in a clinic, we acknowledge some limitations. Detection of *Mtb* DNA by Ultra cannot confirm the presence of viable bacteria, leading to questions about its validity to estimate infectiousness. However, in high-TB settings among untreated symptomatic people, DNA detection likely serves as a reasonable proxy for viability and enables rapid decision making. The detection of environmental *Mtb* DNA from within sampling booths also raises the possibility of aerosol detection measurement error. To limit the potential effect of this bias, our primary analysis excluded all sampling sessions with positive environmental samples. We also reported sensitivity analysis results using a more stringent approach, which showed similar results.

The opportunity to measure AMD in primary care might offer novel insights into the infectiousness of individuals being investigated for TB. For instance, there is a need to identify and characterize “super spreaders,” who disproportionately contribute to community transmission [38], since their effective management can dramatically reduce propagation. Super spreaders have been well described in other respiratory infectious diseases [39], but few studies have evaluated this phenomenon in TB despite many descriptive case series [40, 41]. By using the sputum to aerosol IS*1081/*IS*6110* C_T_ ratio, we were able to identify participants who could expel AMD more effectively than others, a feature that may be consistent with greater infectiousness. While the significance of this finding remains uncertain, the importance of identifying, characterizing, and intervening in this group warrants further investigation.

We conclude that AMD detection by clinical staff is feasible in a clinical setting with THOR. This portable device could be used to characterize infectiousness in more diverse settings and in previously unevaluated groups, particularly those with minimal sputum bacillary load or limited chest x-ray abnormalities. Our results support the findings from previous studies using more complex samplers and raise the possibility of sex influencing the generation of *Mtb* aerosols. They also provide some support to the possibility of community transmission originating from people with negative sputum *Mtb* results, and they offer a potential tool to screen more people for TB transmission risk, irrespective of their clinical status.

## Supplementary Material

ofaf593_Supplementary_Data

## Supplementary Data

Supplementary materials are available at *Open Forum Infectious Diseases* online. Consisting of data provided by the authors to benefit the reader, the posted materials are not copyedited and are the sole responsibility of the authors, so questions or comments should be addressed to the corresponding author.
