# Correction to: Detection of Aerosolized *Mycobacterium tuberculosis* DNA From Adults Being Investigated for Pulmonary Tuberculosis via an Electrostatic Sampler in a South African Primary Care Setting

**DOI:** 10.1093/ofid/ofag520

**Published:** 2026-08-24

This is a correction to: Jay Achar, Rouxjeane Venter, Jamie van Schalkwyk, Zandile Booi, Zama Mahlobo, Zaida Palmer, Nuno Rufino de Sousa, Knut Lönnroth, James A Seddon, Antonio Gigliotti Rothfuchs, Grant Theron, Detection of Aerosolized *Mycobacterium tuberculosis* DNA From Adults Being Investigated for Pulmonary Tuberculosis via an Electrostatic Sampler in a South African Primary Care Setting, *Open Forum Infectious Diseases*, Volume 12, Issue 10, October 2025, ofaf593, https://doi.org/10.1093/ofid/ofaf593

In the originally published version of this manuscript, the values in the row marked “≤Low/negative” under the variable “Sputum Ultra category” in Table 2 were incorrect.

These values have been corrected to read “15 (23)” and “51 (77)” instead of “7 (22)” and “25 (78)”.

