# Supplementary material for: Detection of Aerosolized Mycobacterium tuberculosis DNA From Adults Being Investigated for Pulmonary Tuberculosis via an Electrostatic Sampler in a South African Primary Care Setting
Source: Open Forum Infect Dis. 2025 Sep 24;12(10):ofaf593. doi: 10.1093/ofid/ofaf593 (PMC12534728; doi:10.1093/ofid/ofaf593)
Supplement: ofaf593_Supplementary_Data [file ofaf593_supplementary_data.docx]

# Supplementary table 1

Availability of environmental control and environmental control results by participant characteristics

|  | **Environment sampling performed** | | | **Environment result when available** | | |
| --- | --- | --- | --- | --- | --- | --- |
|  | **Yes**  N = 137*^a^* | **No**  N = 41*^a^* | **p-value***^b^* | ***Mtb* detected**  N = 41*^a^* | ***Mtb* not detected**  N = 96*^a^* | **p-value***^2^* |
| **Age (yrs)** | 37 (30, 47) | 36 (29, 42) | 0.6 | 40 (31, 46) | 36 (29, 48) | 0.2 |
| **Sex** |  |  | >0.9 |  |  | 0.9 |
| Female | 59 (43%) | 18 (44%) |  | 18 (44%) | 41 (43%) |  |
| Male | 78 (57%) | 23 (56%) |  | 23 (56%) | 55 (57%) |  |
| **Body Mass Index** | 21.3 (19.5, 25.9) | 21.8 (18.3, 24.0) | 0.7 | 22.0 (19.8, 28.2) | 21.0 (18.9, 24.8) | 0.11 |
| **Body Mass Index (kg/m^2^)** |  |  | 0.2 |  |  | 0.12 |
| ≤18.5 | 24 (18%) | 11 (27%) |  | 4 (9.8%) | 20 (21%) |  |
| >18.5 | 113 (82%) | 30 (73%) |  | 37 (90%) | 76 (79%) |  |
| **HIV status*^c^*** |  |  | 0.6 |  |  | 0.9 |
| Negative | 78 (57%) | 25 (61%) |  | 23 (56%) | 55 (57%) |  |
| Positive | 59 (43%) | 16 (39%) |  | 18 (44%) | 41 (43%) |  |
| **History of TB*^d^*** |  |  | 0.2 |  |  | 0.5 |
| No | 94 (69%) | 24 (59%) |  | 30 (73%) | 64 (67%) |  |
| Yes | 43 (31%) | 17 (41%) |  | 11 (27%) | 32 (33%) |  |
| **Smoking history** |  |  | 0.4 |  |  | 0.2 |
| Never | 61 (45%) | 23 (56%) |  | 23 (56%) | 38 (40%) |  |
| Ex-smoker | 21 (15%) | 4 (9.8%) |  | 5 (12%) | 16 (17%) |  |
| Current smoker | 55 (40%) | 14 (34%) |  | 13 (32%) | 42 (44%) |  |
| **Total cough count** | 82 (58, 105) | 53 (47, 83) | 0.003 | 77 (58, 104) | 82 (59, 106) | 0.9 |
| **Sputum Xpert Ultra C_Tmin_*^e^*** | 19 (17, 24) | 18 (18, 20) | 0.6 | 20 (17, 26) | 18 (17, 24) | 0.5 |
| Unavailable^f^ | 62 | 1 |  | 25 | 37 |  |
| **Sputum Xpert Ultra result** |  |  | <0.001 |  |  | 0.3 |
| Mtb detected | 86 (63%) | 40 (98%) |  | 23 (56%) | 63 (66%) |  |
| Mtb not detected | 51 (37%) | 1 (2.4%) |  | 18 (44%) | 33 (34%) |  |
| **Sputum Xpert Ultra semi-quantitative result** |  |  | <0.001 |  |  | 0.2 |
| High | 36 (26%) | 21 (51%) |  | 6 (15%) | 30 (31%) |  |
| Medium | 11 (8.1%) | 13 (32%) |  | 4 (10%) | 7 (7.3%) |  |
| Low | 20 (15%) | 2 (4.9%) |  | 4 (10%) | 16 (17%) |  |
| Very Low | 10 (7.4%) | 4 (9.8%) |  | 4 (10%) | 6 (6.3%) |  |
| Trace | 8 (5.9%) | 0 (0%) |  | 4 (10%) | 4 (4.2%) |  |
| Negative | 51 (38%) | 1 (2.4%) |  | 18 (45%) | 33 (34%) |  |
| Missing | 1 | 0 |  | 1 | 0 |  |
| **Karnofsky score** |  |  | 0.002 |  |  | 0.3 |
| 50 | 1 (0.7%) | 0 (0%) |  | 0 (0%) | 1 (1.0%) |  |
| 60 | 5 (3.6%) | 1 (2.4%) |  | 3 (7.3%) | 2 (2.1%) |  |
| 70 | 6 (4.4%) | 4 (9.8%) |  | 1 (2.4%) | 5 (5.2%) |  |
| 80 | 32 (23%) | 22 (54%) |  | 6 (15%) | 26 (27%) |  |
| 90 | 72 (53%) | 12 (29%) |  | 23 (56%) | 49 (51%) |  |
| 100 | 21 (15%) | 2 (4.9%) |  | 8 (20%) | 13 (14%) |  |
| *^a^*Median (Q1, Q3); n (%) | | | | | | |
| *^b^*Wilcoxon rank sum test; Pearson's Chi-squared test; Fisher's exact test | | | | | | |
| *^c^*HIV = Human immunodeficiency virus | | | | | | |
| *^d^*TB = tuberculosis | | | | | | |
| *^e^*C_Tmin_ = Minimum cycle threshold | | | | | | |
| *^f^Unavailable C_Tmin_=* All missing C_Tmin_ values were from trace or negative sputum Ultra results | | | | | | |

# Supplementary table 2

Sputum Xpert Ultra semi-quantitative result by participant characteristics

|  | **Sputum Xpert Ultra semi-quantitation** | | | | | |
| --- | --- | --- | --- | --- | --- | --- |
|  | **High**  N = 51*^a^* | **Medium**  N = 20*^a^* | **Low**  N = 18*^a^* | **Very Low**  N = 10*^a^* | **Trace**  N = 4*^a^* | **Negative**  N = 34*^a^* |
| **Age (yrs)** | 38 (27, 46) | 38 (32, 51) | 35 (23, 39) | 34 (29, 49) | 43 (32, 50) | 34 (30, 44) |
| **Sex** |  |  |  |  |  |  |
| Female | 18 (35%) | 8 (40%) | 6 (33%) | 2 (20%) | 3 (75%) | 22 (65%) |
| Male | 33 (65%) | 12 (60%) | 12 (67%) | 8 (80%) | 1 (25%) | 12 (35%) |
| **Body Mass Index (kg/m^2^)** | 21.0 (18.2, 23.9) | 20.6 (18.2, 23.3) | 21.8 (19.7, 25.1) | 20.3 (18.4, 21.4) | 19.4 (16.7, 22.8) | 24.5 (20.5, 29.8) |
| ≤18.5 | 13 (25%) | 8 (40%) | 0 (0%) | 3 (30%) | 2 (50%) | 5 (15%) |
| >18.5 | 38 (75%) | 12 (60%) | 18 (100%) | 7 (70%) | 2 (50%) | 29 (85%) |
| **HIV status*^b^*** |  |  |  |  |  |  |
| Negative | 33 (65%) | 12 (60%) | 9 (50%) | 7 (70%) | 2 (50%) | 17 (50%) |
| Positive | 18 (35%) | 8 (40%) | 9 (50%) | 3 (30%) | 2 (50%) | 17 (50%) |
| **History of TB*^c^*** |  |  |  |  |  |  |
| No | 33 (65%) | 10 (50%) | 17 (94%) | 4 (40%) | 3 (75%) | 21 (62%) |
| Yes | 18 (35%) | 10 (50%) | 1 (5.6%) | 6 (60%) | 1 (25%) | 13 (38%) |
| **Smoking history** |  |  |  |  |  |  |
| Never | 21 (41%) | 8 (40%) | 12 (67%) | 2 (20%) | 2 (50%) | 16 (47%) |
| Ex-smoker | 11 (22%) | 1 (5.0%) | 2 (11%) | 1 (10%) | 0 (0%) | 5 (15%) |
| Current smoker | 19 (37%) | 11 (55%) | 4 (22%) | 7 (70%) | 2 (50%) | 13 (38%) |
| **Total cough count** | 66 (51, 102) | 67 (50, 101) | 73 (49, 107) | 87 (50, 97) | 85 (46, 108) | 75 (52, 106) |
| **Sputum Xpert Ultra C_Tmin_*^d^*** | 18 (17, 18) | 19 (19, 20) | 24 (21, 25) | 29 (29, 30) | - | - |
| Unavailable*^e^* | 0 | 0 | 0 | 0 | 4 | 34 |
| **Sputum Xpert Ultra result** |  |  |  |  |  |  |
| *Mtb* detected | 51 (100%) | 20 (100%) | 18 (100%) | 10 (100%) | 4 (100%) | 0 (0%) |
| *Mtb* not detected | 0 (0%) | 0 (0%) | 0 (0%) | 0 (0%) | 0 (0%) | 34 (100%) |
| **Karnofsky score** |  |  |  |  |  |  |
| 50 | 1 (2.0%) | 0 (0%) | 0 (0%) | 0 (0%) | 0 (0%) | 0 (0%) |
| 60 | 2 (3.9%) | 0 (0%) | 1 (5.6%) | 0 (0%) | 0 (0%) | 0 (0%) |
| 70 | 5 (9.8%) | 2 (10%) | 1 (5.6%) | 0 (0%) | 0 (0%) | 1 (2.9%) |
| 80 | 22 (43%) | 10 (50%) | 3 (17%) | 5 (50%) | 1 (25%) | 7 (21%) |
| 90 | 21 (41%) | 7 (35%) | 11 (61%) | 4 (40%) | 2 (50%) | 16 (47%) |
| 100 | 0 (0%) | 1 (5.0%) | 2 (11%) | 1 (10%) | 1 (25%) | 10 (29%) |
| **Reported cough** |  |  |  |  |  |  |
| No | 2 (3.9%) | 0 (0%) | 1 (5.6%) | 0 (0%) | 0 (0%) | 3 (8.8%) |
| Yes | 49 (96%) | 20 (100%) | 17 (94%) | 10 (100%) | 4 (100%) | 31 (91%) |
| **Reported fever** |  |  |  |  |  |  |
| No | 35 (69%) | 12 (60%) | 14 (78%) | 6 (60%) | 2 (50%) | 25 (74%) |
| Yes | 16 (31%) | 8 (40%) | 4 (22%) | 4 (40%) | 2 (50%) | 9 (26%) |
| **Reported weight-loss** |  |  |  |  |  |  |
| No | 5 (9.8%) | 3 (15%) | 3 (17%) | 1 (10%) | 2 (50%) | 17 (50%) |
| Yes | 46 (90%) | 17 (85%) | 15 (83%) | 9 (90%) | 2 (50%) | 17 (50%) |
| **Reported night-sweats** |  |  |  |  |  |  |
| No | 12 (24%) | 6 (30%) | 7 (39%) | 3 (30%) | 1 (25%) | 16 (47%) |
| Yes | 39 (76%) | 14 (70%) | 11 (61%) | 7 (70%) | 3 (75%) | 18 (53%) |
| *^a^*Median (Q1, Q3); n (%) | | | | | | |
| *^b^*HIV = Human immunodeficiency virus | | | | | | |
| *^c^*TB = tuberculosis | | | | | | |
| *^d^*C_Tmin_ = Minimum cycle threshold | | | | | | |
| *^e^*Unavailable = *rpoB* Ct values are not reported by Xpert Ultra for trace or negative results | | | | | | |

# Supplementary table 3

Aerosolized *Mtb* DNA detection diagnostic performance by sputum *Mtb* DNA burden

|  | **Aerosol *Mtb* DNA detected** | | **Sensitivity***^b^* | | **Specificity***^b^* |
| --- | --- | --- | --- | --- | --- |
|  | **Yes**  N = 56*^a^* | **No**  N = 81*^a^* |  | |  |
| **Sputum Xpert Ultra result** |  |  |  | |  |
| *Mtb* detected | 48 | 55 | 46.6 (42.5, 50.7) | |  |
| *Mtb* not detected | 8 | 26 |  | | 76.5 (70.4, 82.5) |
| **Sputum Xpert Ultra semi-quantitative result** |  |  |  | |  |
| High | 29 | 22 | 56.9 (51.1, 62.7) | |  |
| Medium | 12 | 8 | 60.0 (50.9, 69.1) | |  |
| Low | 4 | 14 | 22.2 (14.0, 30.4) | |  |
| Very Low | 3 | 7 | 30.0 (17.9, 42.1) | |  |
| Trace | 0 | 4 | 0 (0, 0) | |  |
| Negative | 8 | 26 |  | | 76.5 (70.4, 82.5) |
| *^a^*n (%) | | | |  |  |
| *^b^* % (95% Confidence Interval) | | | |  |  |

# Supplementary table 4

Aerosol *Mtb* Ultra semi-quantitative result in positive aerosol specimens

|  | **N = 56***^a^* |
| --- | --- |
| **Aerosol Xpert Ultra semi-quantitative result** |  |
| High | 0 (0%) |
| Medium | 0 (0%) |
| Low | 3 (5%) |
| Very Low | 14 (25%) |
| Trace | 39 (70%) |
| *^a^*n (%) | |

# Supplementary table 5

Crude associations between participant characteristic and aerosolized *Mtb* DNA detection by sensitivity analysis cohort

|  | **Cohort 1**  **(n = 137)** | | | **Cohort 2**  **(n = 96)** | | | **Cohort 3**  **(n = 109)** | | | **Cohort 4**  **(n = 150)** | | |
| --- | --- | --- | --- | --- | --- | --- | --- | --- | --- | --- | --- | --- |
|  | **RR***^a^* | **95% CI***^a^* | **p-value** | **RR***^a^* | **95% CI***^a^* | **p-value** | **RR***^a^* | **95% CI***^a^* | **p-value** | **RR***^a^* | **95% CI***^a^* | **p-value** |
| **Age (yrs)** | 1.01 | 0.99, 1.03 | 0.3 | 1.01 | 0.99, 1.04 | 0.4 | 1.01 | 0.99, 1.03 | 0.4 | 1.01 | 0.99, 1.02 | 0.3 |
| **Sex** |  |  |  |  |  |  |  |  |  |  |  |  |
| Female | — | — |  | — | — |  | — | — |  | — | — |  |
| Male | 1.74 | 1.09, 2.75 | 0.019 | 2.34 | 1.10, 4.99 | 0.027 | 1.57 | 0.93, 2.66 | 0.089 | 1.47 | 1.01, 2.15 | 0.045 |
| **Body Mass Index** | 1.02 | 0.98, 1.06 | 0.3 | 1.02 | 0.96, 1.08 | 0.5 | 1.02 | 0.97, 1.06 | 0.5 | 1.01 | 0.98, 1.05 | 0.3 |
| **Body Mass Index (kg/m^2^)** |  |  |  |  |  |  |  |  |  |  |  |  |
| ≤18.5 | — | — |  | — | — |  | — | — |  | — | — |  |
| >18.5 | 0.97 | 0.60, 1.56 | 0.9 | 1.26 | 0.55, 2.92 | 0.6 | 1.43 | 0.69, 2.97 | 0.3 | 1.07 | 0.69, 1.66 | 0.8 |
| **HIV status*^b^*** |  |  |  |  |  |  |  |  |  |  |  |  |
| Negative | — | — |  | — | — |  | — | — |  | — | — |  |
| Positive | 0.78 | 0.51, 1.20 | 0.3 | 0.43 | 0.20, 0.91 | 0.027 | 0.46 | 0.25, 0.84 | 0.012 | 0.75 | 0.51, 1.10 | 0.14 |
| **History of TB*^c^*** |  |  |  |  |  |  |  |  |  |  |  |  |
| No | — | — |  | — | — |  | — | — |  | — | — |  |
| Yes | 0.92 | 0.60, 1.42 | 0.7 | 1.05 | 0.55, 2.00 | 0.9 | 0.88 | 0.52, 1.51 | 0.6 | 0.85 | 0.58, 1.25 | 0.4 |
| **Smoking history** |  |  |  |  |  |  |  |  |  |  |  |  |
| Never | — | — |  | — | — |  | — | — |  | — | — |  |
| Ex-smoker | 1.14 | 0.64, 2.05 | 0.7 | 1.58 | 0.67, 3.76 | 0.3 | 1.11 | 0.56, 2.23 | 0.8 | 1.03 | 0.61, 1.73 | >0.9 |
| Current smoker | 1.04 | 0.67, 1.63 | 0.9 | 1.41 | 0.68, 2.90 | 0.4 | 1.06 | 0.62, 1.80 | 0.8 | 0.97 | 0.66, 1.42 | 0.9 |
| **Total cough count** | 0.99 | 0.99, 1.00 | 0.038 | 1.0 | 0.99, 1.00 | 0.3 | 1.00 | 1.0, 1.01 | 0.7 | 1.00 | 0.99, 1.00 | 0.4 |
| **Sputum Xpert Ultra C_Tmin_*^d^*** | 0.95 | 0.93, 0.98 | 0.003 | 0.96 | 0.92, 1.00 | 0.031 | 0.98 | 0.95, 1.01 | 0.13 | 0.97 | 0.95, 1.00 | 0.019 |
| **Sputum Xpert Ultra result** |  |  |  |  |  |  |  |  |  |  |  |  |
| *Mtb* detected | — | — |  | — | — |  | — | — |  | — | — |  |
| *Mtb* not detected | 0.50 | 0.26, 0.96 | 0.038 | 0.61 | 0.29, 1.28 | 0.2 | 0.75 | 0.43, 1.29 | 0.3 | 0.66 | 0.41, 1.07 | 0.093 |
| **Sputum Xpert Ultra category** |  |  |  |  |  |  |  |  |  |  |  |  |
| ≥ medium | — | — |  | — | — |  | — | — |  | — | — |  |
| ≤ low | 0.38 | 0.19, 0.76 | 0.006 | 0.32 | 0.12, 0.84 | 0.021 | 0.50 | 0.25, 0.97 | 0.040 | 0.51 | 0.30, 0.87 | 0.013 |
| negative | 0.41 | 0.21, 0.78 | 0.006 | 0.44 | 0.21, 0.92 | 0.030 | 0.59 | 0.34, 1.03 | 0.061 | 0.56 | 0.34, 0.90 | 0.018 |
| **Karnofsky score** |  |  |  |  |  |  |  |  |  |  |  |  |
| >80 | — | — |  | — | — |  | — | — |  | — | — |  |
| ≤80 | 1.44 | 0.96, 2.16 | 0.080 | 1.70 | 0.93, 3.11 | 0.084 | 1.48 | 0.93, 2.37 | 0.10 | 1.31 | 0.93, 1.85 | 0.13 |
| **Reported cough** |  |  |  |  |  |  |  |  |  |  |  |  |
| No | — | — |  | — | — |  | — | — |  | — | — |  |
| Yes | 2.52 | 0.41, 15.5 | 0.3 | 1.54 | 0.25, 9.30 | 0.6 | 1.97 | 0.33, 11.8 | 0.5 | 2.83 | 0.46, 17.3 | 0.3 |
| **Reported fever** |  |  |  |  |  |  |  |  |  |  |  |  |
| No | — | — |  | — | — |  | — | — |  | — | — |  |
| Yes | 0.60 | 0.35, 1.01 | 0.056 | 0.20 | 0.05, 0.79 | 0.022 | 0.37 | 0.16, 0.86 | 0.021 | 0.63 | 0.40, 0.99 | 0.046 |
| **Reported weight-loss** |  |  |  |  |  |  |  |  |  |  |  |  |
| No | — | — |  | — | — |  | — | — |  | — | — |  |
| Yes | 1.35 | 0.77, 2.35 | 0.3 | 1.23 | 0.59, 2.56 | 0.6 | 1.12 | 0.64, 1.95 | 0.7 | 1.20 | 0.76, 1.88 | 0.4 |
| **Reported night-sweats** |  |  |  |  |  |  |  |  |  |  |  |  |
| No | — | — |  | — | — |  | — | — |  | — | — |  |
| Yes | 1.12 | 0.72, 1.76 | 0.6 | 0.98 | 0.52, 1.85 | >0.9 | 0.87 | 0.54, 1.40 | 0.6 | 1.0 | 0.69, 1.43 | >0.9 |
| *^a^*RR = Risk Ratio, CI = Confidence Interval calculated with robust Standard Errors | | | | | | | | | | | | |
| *^b^*HIV = Human immunodeficiency virus | | | | | | | | | | | | |
| *^c^*TB = tuberculosis | | | | | | | | | | | | |
| *^d^*C_Tmin_ = Minimum cycle threshold | | | | | | | | | | | | |

# Supplementary figure 1


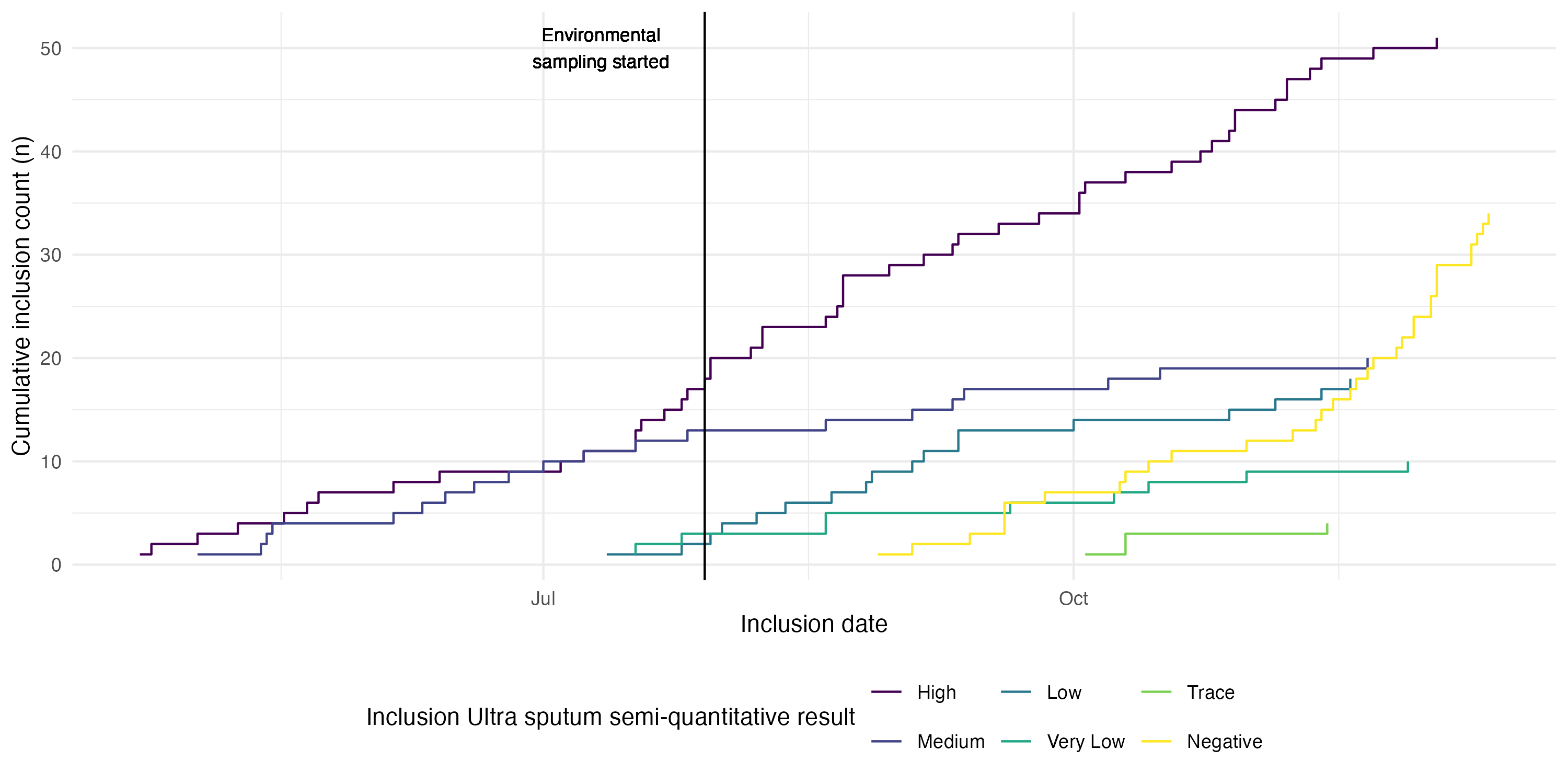


**Supplementary figure 1**. Study inclusion started on 22nd April 2024 and was restricted to people with positive sputum Ultra tests and with semi-quantitative results high or medium until 12th July 2024, after which all eligible participants were included. Environmental sampling prior to each participant started on 29th July 2024 and continued throughout the remainder of the study.

# Supplementary figure 2


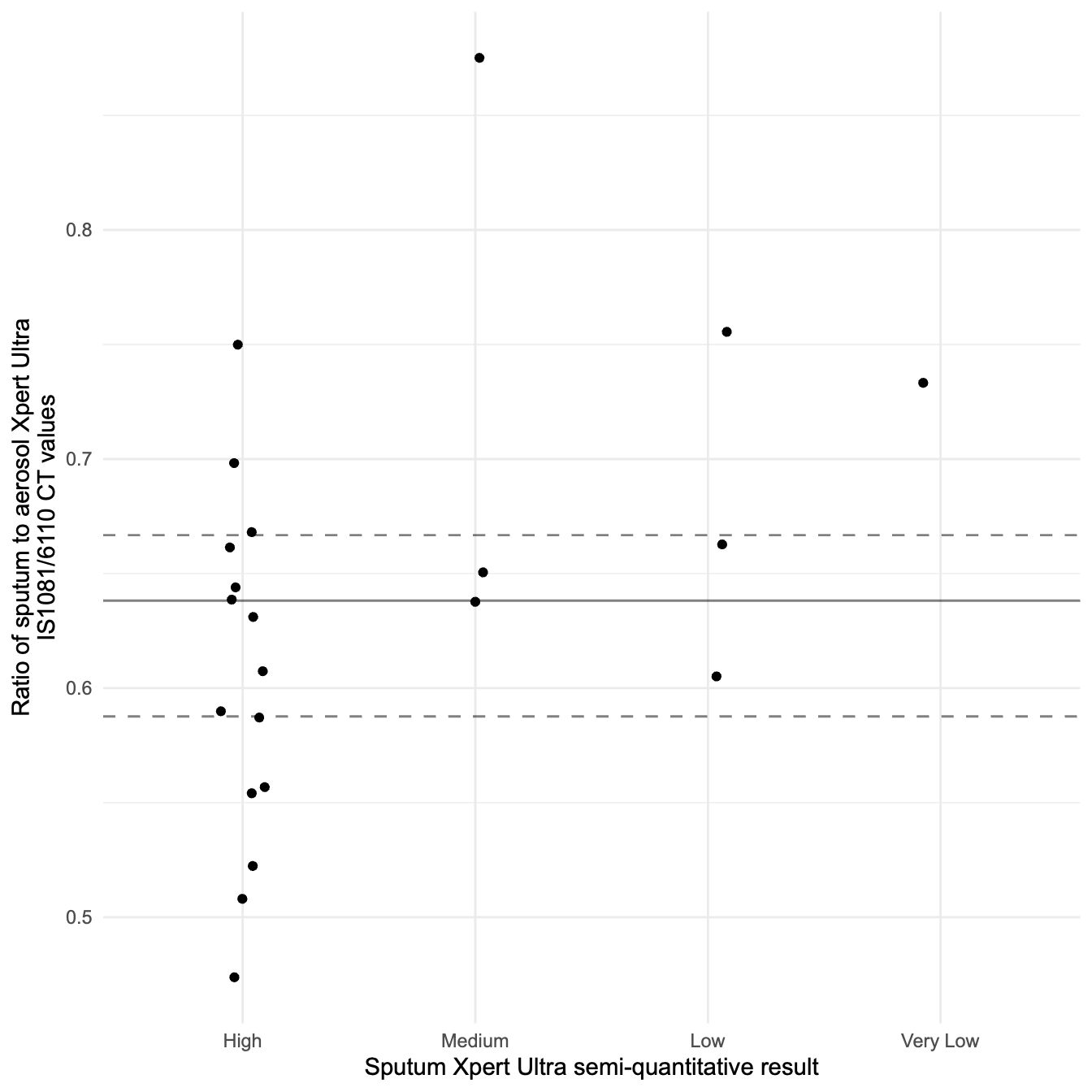


**Supplementary figure 2**. Comparing the ratio of sputum to aerosol Xpert Ultra *IS1081/6110* C_T_ values by sputum Xpert Ultra semi-quantitative result. Ratios from participants in Cohort 2 with positive sputum and positive AMD Ultra (22/96) are shown. The median ratio (black solid line, 0.64) and the inter-quartile range (dotted line, 0.59, 0.67) are shown. The distribution of participants with ratios in the highest quartile appears evenly spread across sputum Ultra semi-quantitative grades. The top 5 ratios range between 0.87 and 0.70.

# Supplementary figure 3

**
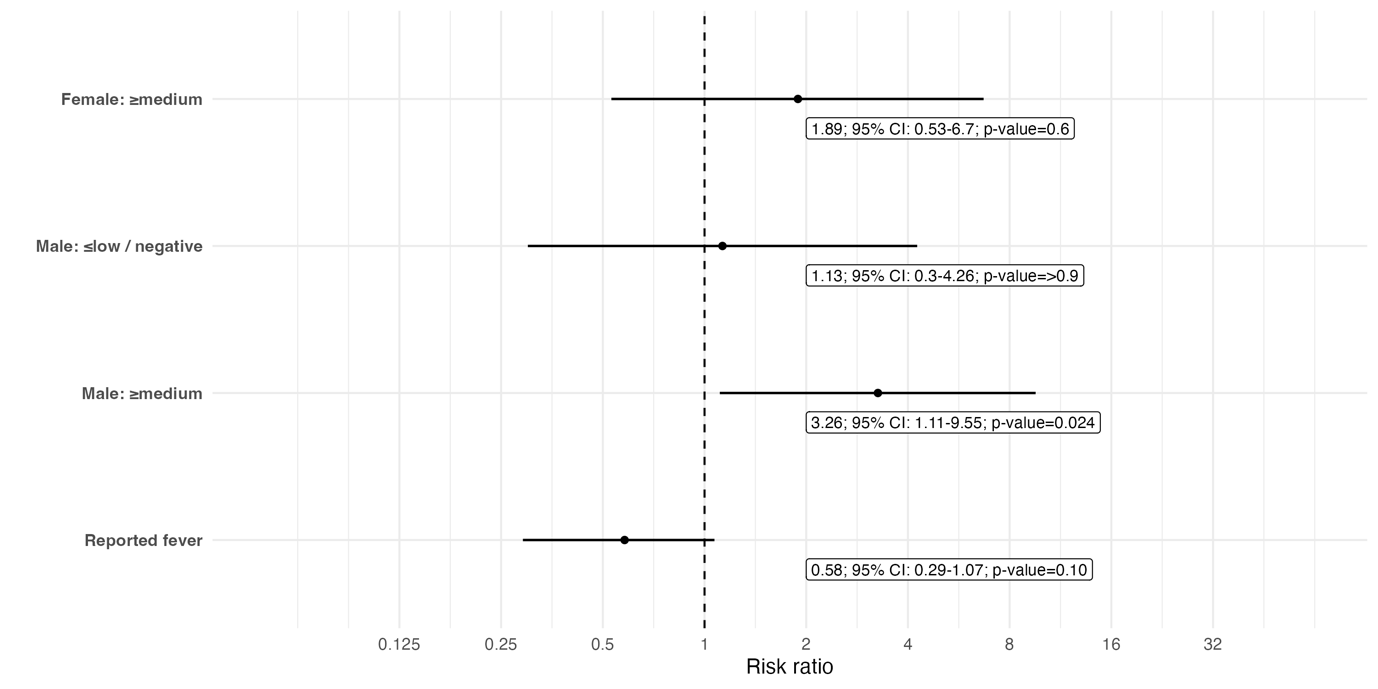
**

**Supplementary figure 3**: Forest plot showing adjusted modified Poisson regression model parameter estimates with corresponding 95% confidence intervals of participant characteristics and aerosolized *M. tuberculosis* DNA detection. The interaction between sex and sputum Ultra semi-quantitative result has been combined to display parameter estimates for each resulting group with the base group being women with ≤ low / negative sputum Ultra semi-quantitative result.

# Supplementary figure 4


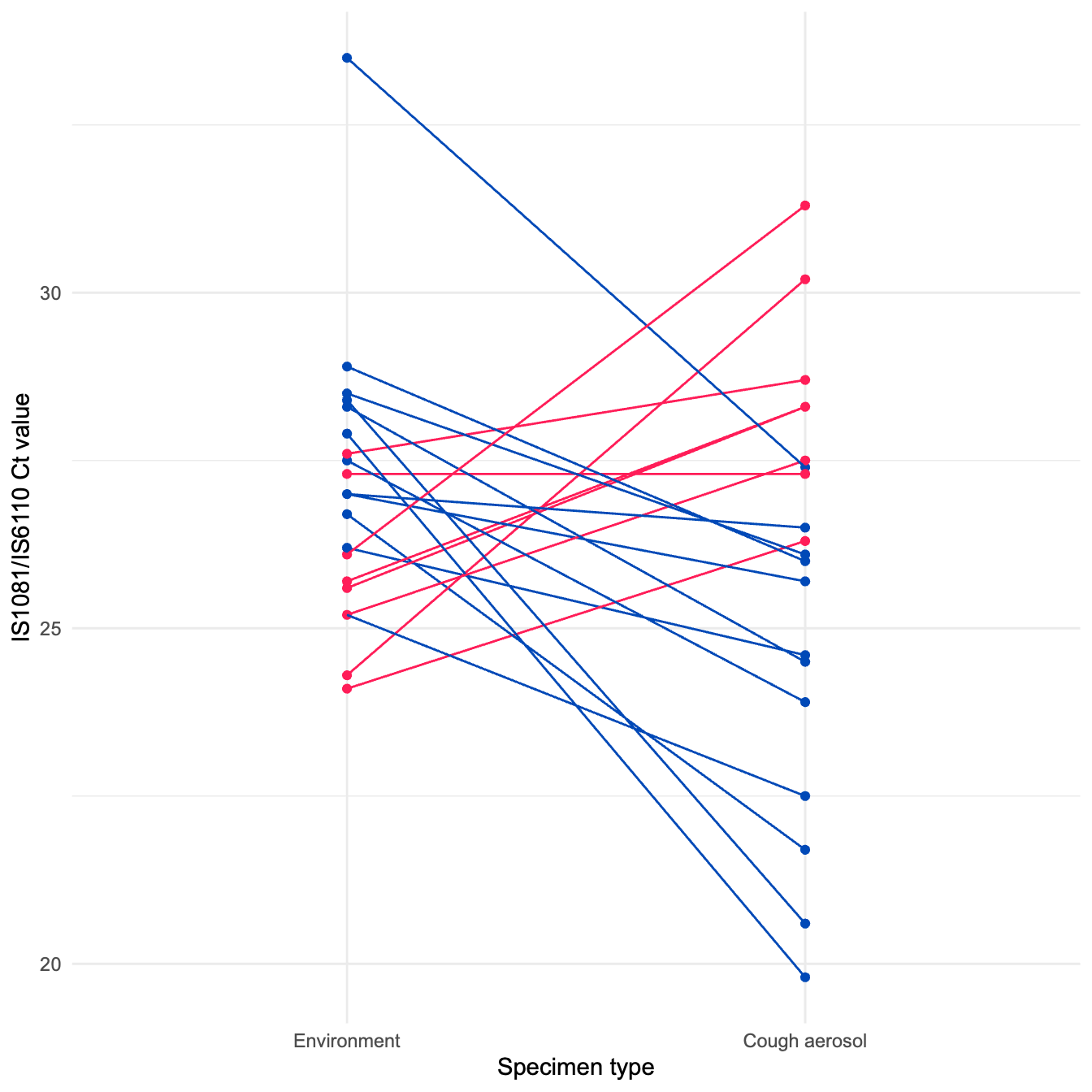


**Supplementary figure 4**: Grouped line plot of 21 participants with a positive environmental aerosolized *M. tuberculosis* DNA (AMD) result and a positive cough AMD result illustrating the difference in *IS1081/IS6119* C_T_ value between the two samples. Pairs in which the cough aerosol C_T_ value is lower (13/21; 62%) (blue) than the corresponding environmental C_T_ value represent sessions where greater AMD is detected after the participant provided their cough sample suggesting cough AMD positivity.
